# The Marvellous
**Histoire du Merveilleux dans les Temps Modernes.* Par Louis Figuier. Paris, 1860.*The Spiritual Magazine.* London, 1860.*The Arcana of Christianity, and various Sermons.* By the Rev. T. L. Harris.


**Published:** 1861-01

**Authors:** 


### MEDICAL CRITIC

AND

### PSYCHOLOGICAL

### JOURNAL.

JANUARY, 1861.

### Art. I.—THE MARVELLOUS.*

In the last number of the past series of this Journal we briefly reviewed the chief "phenomenal phases," as the Spiritualists say, under which the love of mankind for the marvellous has been displayed in past centuries. We purpose in the present article to consider the manifestations displayed in our own time; and it will not be amiss, in the first place, to recapitulate the actual claims of the Spiritualists, as stated by a prominent brother of the order, who is wishful to "present a brief general statement of the leading phenomenal phases in which, at the present day, Spiritualism is presented to us."

"Before doing so," writes this author (*Spiritual Magazine*, No. 2), "as a preliminary observation necessary to a right understanding of the matter, we would remark, that there are persons in some way peculiarly constituted whose presence appears to furnish conditions requisite to enable spirits to act upon matter, or to manifest their agency in any way cognizable to men. In what this peculiarity consists, whether it be chemical, electrical, magnetic, odylic, or in some combination of these, or in what else, it would lead us too far from our present purpose to consider." (We break into the quotation at this point, in order to call attention to the effects of spiritual intercourse upon English composition.) "At present we would only point out the fact that the presence of one such person at least is necessary in

* *Histoire du Merveilleux dans les Temps Modernes.* Par Louis Figuier. Paris, 1860.

*The Spiritual Magazine.* London, 1860.

*The Arcana of Christianity, and various Sermons.* By the Rev. T. L. Harris.

every circle before any spiritual manifestations can be obtained. Such persons are now technically designated *mediums*."

The most common form of the manifestations, and that which is most easily obtained, is seen in—

"1. *The Rappings, Table-tippings, and other Sounds and Movements of Ponderable Bodies.*—The company assembled place their hands lightly on a table, and if a suitable medium is present, in a short time sounds, like raps or detonations, are heard on the table, the chairs, the walls, or the floor, often varying in power and tone. . . . . At other times, instead of sounds being heard, extraordinary movements of the table are seen, it rising and falling vertically or perpendicularly, and to different elevations off the floor, or sliding along the room first in one direction and then in another, or moving rapidly round it. . . . . On more than one occasion we have seen the table rise from the floor without any contact . . . . no one being nearer than from two to three feet of it. Human beings also have frequently been raised off the floor and floated round the room in the presence of numerous persons.

"2. *Spirit-writings and Spirit-drawings.*— The former of these modes of communication is not unfrequent. Usually, the medium holds a pencil in hand as for writing, and, sometimes immediately, sometimes after a few minutes, the hand goes into involuntary motion, forming letters, words, and sentences, making an intelligible communication or reply to some question, verbal or mental, that has been asked. . . . . With some mediums the hand is simply used mechanically, the medium not having the slightest idea of what is being written; with others this is accompanied by impression as to the immediate word or sentence that is to be written, but no further. I know one medium who sees before him in the air, or upon the table, the word he has to write. . . . . Cases of direct spirit-writing, that is, not requiring the intervention of a mortal hand, are comparatively rare.

"3. *Trance and Trance-speaking.* . . . .—In this state the trancee frequently speaks as from a spirit—sometimes in long and sustained discourse; and even at times in a foreign and (to the trancee) unknown tongue. We have scores of times heard persons of but little education discourse, when in this state, with an amplitude of knowledge which we are sure they did not in themselves possess, and with a logical coherence and power of expression of which in their normal state they were incapable. . . . . This state is similar, if not identical, with that which in the same persons may be induced by mesmerism.

"4. *Clairvoyance and Clairaudience.* . . .

"5. *Luminous Phenomena* are sometimes seen at spiritual *séances.* They are usually described as very brilliant; sometimes they appear as stars, or as balls of fire; at other times they shoot, meteor-like, through the apartment, or gleam over the walls, or appear as luminous currents circling round a particular centre, such as the hand of the medium, the pencil with which he is writing, or some object in the room.

"6. *Spiritual Impersonation*, or the representation or reproduction in a medium of the actions and manner, gait, deportment, and other peculiarities which distinguished the actuating spirit in his earth-life.

"7. *Spirit-music.*—A musical instrument, say a harp or an accordion, being held or suspended in the hand of the medium, or of some person near him, tunes are sometimes played on it by invisible agency, often in a very superior manner; sometimes it will be a known and familiar tune, at other times spirit-music will be thus improvised.

"We know persons who often, when alone and unexpectedly, hear delightful music, apparently in the air, resembling, and yet unlike, any other they have heard. . . .

"8. *Visible* and *Tactual Manifestations*, such as the appearance and touch of *spirit hands.*

"9. *Spirit Intercourse by means of the mirror, crystal, and vessel of water.* . . .

"10. *Apparitions of the Departed.* . . .

"11. *Visions and Pre-visions.*

"12. *Dreams.* . . .

"13. *Presentiments.* . . .

"14. *Spirit Influx,* by which ideas and sentiments are infused into the mind. . . .

"15. *Involuntary Utterance,* . . .

"16. *Possession.*—We believe that many persons treated as insane are only so in the same sense as the demoniacs of old." . . .

These quotations afford a sufficient basis of information concerning the alleged facts of spiritualism to enable us to investigate its nature and causes, and we now proceed to consider the whole matter under two heads—first, the physical phenomena of a *séance*; and, secondly, the results of spiritual dictation.

It has been conceded by the Spiritualists, over and over again, that the marvels of the *séance* are of the same nature with those wrought under the names of witchcraft, demoniac possession, mesmerism, &c.; and it follows that the facts and arguments bearing upon the latter will apply to the former also. With this preliminary statement, it is necessary first to take into account the element of fraud that enters into such pretended miracles.

In the accounts of *séances* that are held in private houses, we are usually assured that no fraud has taken place; and the character of the host is frequently adduced as a guarantee for the good faith of the performance. We must, however, take into account that all the tricks of a *séance* could be readily accomplished or surpassed by any of the intelligent gentlemen whose vocation it is to exhibit sleight of hand before the public; and hence, where deception is not only possible, but easy, we want some better security against its occurrence than can be furnished by the presumed integrity of the proprietors of drawing-rooms. *Quis custodiet ipsos custodes?* There have been some very great rogues whose honesty would have been thought above suspicion, until the day when their knavery was detected; there are many owners of ottomans and accordions whom it would not be difficult to dupe; and there are even some individuals not wholly lost to a sense of the pleasures of hoaxing. Upon all these grounds, it is fair to require that every display of spirit influence should bear the test of a close and critical examination—and that even the tables of an entertainer should not be exempted from suspicion and from search. In order to show some of the methods of deception that were practised during the prevalence of mesmerism, we proceed to condense a few pages from the work of M. Morin, entitled, *Du Magnetisme et des Sciences Occultes.*

"About twenty years ago (M. Morin, states) the famous Robert Houdin exhibited a new trick, of his own invention, which he called *the second sight.* It was performed as follows:

"A lad of twelve, being placed at one end of the exhibiting saloon, Houdin, crossing to the other side, requested the spectators to bring to him any objects they might have in their possession. He then questioned the lad about these objects; and received exact descriptions of them, without any hesitation or mistake. The following may serve as a specimen of the dialogue. "What do I hold *with* my hand?" "A piece of money." "Of what value?" "Five francs." "Of whose reign?" "Louis Philippe's." "Of what year?" "1831." "What do I hold *in* my hand?" "A box." "For what purpose?" "A snuff-box." "Of what material?" "Gold." "What is there on the lid?" "A portrait"—and so on. The rivals of Houdin, forced to study his trick, at length discovered and imitated it, until at all the fairs the acrobats joined this second sight to their tumbling, and sometimes, in order to add the attraction of mystery to their performances, pretended to mesmerise the person who described the objects. The secret was eventually published, under the name of *anti-magnetism.* It consists in this, that the manner of putting the question conveys the answer. For instance, they would ask, "What do I hold with my hand?" "What do I hold in my hand?" "What is it that I have in my hand?" "What is it that I hold?" &c. By varying the form of question it is easy to establish a conventional language; such that the first form shall signify a piece of money, the second a watch, the third a ring, and so on. As the spectators only presented things that they were accustomed to carry, a very large number of forms of question was not required; and little dictionaries were published, by which, and by the aid of memory, any two confederates could reproduce the trick of second sight. M. Gaudon, among others, in a *brochure* entitled, *The Second Sight Unveiled,* explains in detail many ingenious stratagems; and pleasantly relates that he practised some of them in the presence of a party of mesmerists, declaring throughout that the performance was but a trick. The mesmerists, however, maintained that his confederate was a clairvoyant; and himself a magnetiser of the first order; and would not believe otherwise until they were shown the exact method of procedure.

"It is now well known that this second sight is but an exercise of ingenuity which does not require any unusual powers, and, notwithstanding the *séances*, somnambulism is nothing else. Whenever the magnetiser knows what the somnambulist ought to do or to say, he is able to prompt him in a conventional language, expressed either by words apparently insignificant, or by a pressure of the hand, or some other method of touch, or even by his manner of walking, or of approaching a seat. You write on a morsel of paper something that you wish to have done by the somnambulist; you give this paper to the magnetiser, who reads it, and who, without saying a word, places it in the hand of the subject. Presently what you have written is executed, and then the paper is shown to the applauding audience. It is but a trick. A touch of the hand has sufficed to indicate the part of the somnambulist. One magnetiser who became fashionable in Paris confessed to one of the most honourable members of the Philanthropico-magnetic Society, that he had 180 distinct methods of touching his subject; and that these were all signs, previously agreed upon, by which to make the subject do those things which the audiences were most accustomed to require. Being reproached for his bad faith, he replied that the magnetic lucidity was so variable, as often to require the assistance of other means.

"Certain magnetisers have devised means of communicating with their subjects, without the aid either of words or of contact. We will cite two examples. A juggler who exhibited the trick of second sight, exhibited also the transmission of sensations. The pretended somnambulist held a glass of water; and the performer announced that in drinking it she should experience the taste of any beverage selected by the audience. The name of the beverage required being written upon paper, the operator, commanding perfect silence, placed himself behind his subject, and out of her sight, neither touching her, nor uttering a word, but making mesmeric passes with extended arms. While thus employed he panted violently, as if exerting an energetic effort of the will. The somnambulist drank; and after a few seconds declared that she had tasted whatever liquid had been specified upon the paper. In this case the loud and varied breathing of the operator was the language which served to indicate the beverage that should be named.

"A friend introduced to me a subject said to be endowed with a most rare faculty. This individual having withdrawn, a card was taken at hazard from a pack, and presented to the operator, who, first looking at it, placed it face downwards upon a sheet of white paper, and made believe to mesmerise it. By this process, he said, it left upon the paper an impress visible to the somnambulist. The card being removed, the operator seated himself on a chair, silent, immovable, and even with closed eyes, so as to remove all possibility of collusion. Then, as previously arranged, the subject was brought into the room, and having examined and smelt the paper, he announced, first the colour of the card, and then the card itself. The operator, after receiving our tribute of applause, informed us that the performance was a trick, requiring neither mesmerism nor clairvoyance. The position of his legs with regard to those of his chair, and of his arms and hands, formed a language sufficiently copious to designate all the cards, and enabled the pretended subject to see, at a single glance, which one had been taken from the pack.

"It may be safely concluded, therefore, that in all cases where the magnetiser knows what is required from the somnambulist, and remains in the room with him, the performance is open to suspicion. Such exhibitions prove nothing; inasmuch as it is impossible to exclude from them opportunities of deception.

"It is also necessary to regard with caution all performances of which the programme has been laid down beforehand, because such may be pre-arranged between the mesmeriser and the subject, who then has only to go through the steps of his part in their order. At one period of the *soirée* he will be insensible, next cataleptic, then ecstatic, and so on. In such cases, to require a variation of the programme will often break down the whole performance. For example, at a *séance* at Vauxhall, there were two brothers ; one magnetiser, and the other subject. The former went through a succession of experiments in his own order, and prefaced each one by a statement of what was about to be done ; but he was generally at a considerable distance from his subject—and out of his hearing—which seemed a sufficient security for good faith. At length he announced that he would draw his brother to himself from one end of the stage to the other ; and the better to display the force of the magnetic attraction, he invited four strong men from the audience to form a barrier between himself and the subject, and to endeavour to arrest his passage. The four men took their places before the subject, whose eyes were bound, and who appeared to see nothing, although this had not been ascertained with proper care. The audience awaited with impatience the signal for bringing the attractive force into play. At this moment I advanced with an air of mystery to the magnetiser, took him by the hand, and drew him away from the position in which he had placed himself, inviting him to exercise his attractive force from his new station. We were so placed that the subject could reach us, in a direct line, without coming into contact with the four men. The magnetiser was evidently opposed to my proposition, but having announced that he had power to attract his subject at pleasure, he could not refuse to try an experiment more easy than that which he had announced; the distance being less, and there being no obstacles in the way. He set himself to magnetise with great energy, blowing like a porpoise, and probably expecting that the subject, hearing his breath, would be warned of his change of place; but, unfortunately for him, the noise of the audience drowned that of his breathing. The subject, thinking all prepared, started as if nothing had been changed. Using his fists vigorously, he soon dispersed the four men, and continued his course in a direct line to the place where he expected to find the magnetiser—and then stopped, resting as one who had fulfilled his task, and not appearing to care the least in the world to seek for him who was said to be his centre of attraction. He had not felt, therefore, the force said to be directed towards him, and had yielded to an imaginary attraction; or rather, there had been neither attraction nor magnetism in the case, but simply a concerted programme between two individuals, of such easy tricks that no juggler would venture to exhibit them, although the public regarded them with wonder when presented under the name of mesmerism. The experiment of attraction had been accepted and admired for many nights; and yet one single precaution was sufficient to reduce it to its real value.

"In many exhibitions of clairvoyance, the eyes of the somnambulist are bandaged, and it is said that he can derive no aid from them. Those who have observed such exhibitions carefully, will not be slow to perceive the insufficiency of this precaution. If the somnambulist can name correctly and immediately the objects presented to him, it may be concluded that he can see them. But this is seldom the case. Usually he takes the object in his hands, feels it, and endeavours to form an idea of it by the aid of touch; then conveys it to his forehead and his nostrils—complains of fatigue, and requires the intervention of the magnetiser to give him a fresh dose of fluid. During these preliminaries, if the magnetiser himself can see the object in question, it is clear that the experiment must go for nothing, because by a word, a gesture, or by some of the contrivances described above, he has the means of conveying information to his subject. But suppose that, not attempting to conceal suspicion of the mesmeriser, such precautions are taken as to prevent him from either seeing the object or conveying information, then the somnambulist, by dint of grimaces and contortions, may displace the bandage, and by holding the object in a certain direction, as on his chest, may get such a glimpse of it that, in the case of a writing, he may decipher one or two words. This result, which many persons think wonderful, is altogether ridiculous.

"I was assured that a very famous somnambulist could read through many sheets of paper; and I made one trial of his powers. His eyes were not bandaged, and were not even completely closed. I gave him a book which chanced to be at hand, and which was probably unknown to him. He asked me through how many pages he should read, and I answered, "Twenty." He opened the book at hazard, and applied it to his forehead with many contortions, then took a pencil, and wrote a line upon the book, saying that it would be found twenty pages further on. The leaves were turned, and the line found, not after twenty pages, but after ten, at a place corresponding to that on which he had written. Was this clairvoyance? It is possible,—and yet it may be doubted: the somnambulist, in placing the book upon his forehead, had an opportunity of glancing rapidly at a line somewhere, and in order to be certain that he had not read in the ordinary manner, he should have been prevented from touching the book.

"In order to give proof of clairvoyance, or of a power of seeing through opaque bodies, it would be easy to take precautions that would leave nothing to be desired. For example, instead of a bandage, use a metallic mask, or even free the somnambulist altogether from such fatiguing restraints, and interpose sheets of paper between himself and the object—or close his eyes with the fingers, and place the object above his eyebrows. But when it has been proposed to Mdlle. Pigeaire, and to other somnambulists vaunted for their clairvoyance, to submit to these precautions, they have uniformly refused to do so, and have only consented to perform under such conditions that trickery, being possible, was to be suspected.

"During a *séance*, there are very few people who can control themselves sufficiently to listen in silence. Most frequently, the audience will talk with the somnambulist, will rectify errors as they are committed, and will approve correct answers; all of them proceedings that must facilitate the task. Often the manner in which a question is put is sufficient to suggest the response. Finally, the audience are astonished by a performance to which these aids have been afforded; they forget the inaccuracies and mistakes, and remember only what has been rightly said, without reflecting that they have themselves supplied the little truth that they have heard. Any person whatever, not clairvoyant, but guessing—having errors constantly corrected, and having ingenuity to frame fresh answers, could not fail to be right sometimes; and could officiate at consultations precisely similar to the bulk of those that are held daily by the somnambulists.

"Whoever wishes to be certain that the responses, as they should do, emanate entirely from the somnambulist and are not suggested piecemeal, should carefully refrain from any remark or observation. But the somnambulists do not like the persons who proceed thus. They say that such audiences set them at defiance, freeze and take away their powers. And they say truly; for, as most of them are not at all clairvoyant, and only accomplish tricks of address by making people chatter, if the audience remain silent, the performers can no longer play their little parts."

This lengthy citation from M. Morin, while it cannot be regarded as conclusive against the pretensions of all mesmerists, is sufficient to establish that fraudulent practices were the rule, rather than the exception, in the public exhibitions of their art. It will be in the recollection of our readers that Sir J. Forbes instituted a searching examination into the performances of every professing clairvoyant who came before the London public; and that in every single case, without exception, he either detected and exposed trickery, or else produced complete failure by the employment of precautions that rendered trickery impossible. It is well known also, that a bank-note of large amount remained, for a considerable time, as a prize for any clairvoyant who could decipher its number through two or three thicknesses of paper: a feat much more easy than many of those which they professed to accomplish daily. We believe that no attempt was ever made to obtain the note in question—and, at all events, no such attempt was successful.

From these facts we infer that, as all the physical phenomena of so-called spiritual intercourse could be produced with great facility by simple mechanical contrivances, it is reasonable to suppose that they are so produced in the majority of instances. Mr. E. Delaware Lewis, in a recent number of *Once a Week*, describes a spiritualist *séance* at which he was present, and speaks of the contrivances by which the effects were produced as being too transparently fraudulent to impose upon any but the most credulous of mankind. That the desire to believe and to wonder does very seriously affect the faculties of observation and judgment in many cases, is a truth too familiar to be called in question; and it has lately received a remarkable illustration in a paper called *Stranger than Fiction*; the strangest thing about which was, that it found admission into a publication so respectable as the *Cornhill Magazine*. The writer, supposing him to write in good faith, has so jumbled together possible occurrences with opinions and with accounts of his own emotional state, that no judgment whatever can be based upon the resulting medley.

Offering himself to the public as a narrator of events which he admits to be scarcely credible, he yet exhibits in every line of his composition the most absolute ignorance of the ordinary sources of self-deception. He relates, for instance, that Mr. Home, the medium, went *floating about in a darkened room;* and that the audience could "judge by his voice of the altitude and distance he had attained." It is humiliating to think that any one, in a position to be described as a personal friend by Mr. Thackeray, can have composed such rigmarole as this, or can be ignorant that a modulation of the voice, sufficient to produce erroneous impressions with regard to the altitude and distance of the speaker, is the easiest of all possible performances. On the face of the record there is not a tittle of evidence that Mr. Home floated about the room at all; and the facts appear to be that a dark outline, resembling that of a human figure, was seen to cross and recross between the spectators and the window, that something, stated to be a foot, touched somebody's chair, and that Mr. Home carried on a conversation, in the course of which the tones of his voice varied from time to time. The writer does not appear to have a suspicion that these events are a sorry foundation for the very splendid hypothesis that has been raised upon them.

It is curious, but in the *Spiritual Magazine* for June, 1860, there is an account of two evenings with Mr. Home; and the tricks recorded are in all essentials identical with those described in the *Cornhill Magazine.* The narrative given in the latter, however, is much more highly coloured than that in the former; and a comparison between them leads irresistibly to one of two conclusions. Either Mr. Home's range of performance is limited, and the pursuits of his familiar spirits are remarkably monotonous, or else the two narratives refer to the same events. In the latter case, Mr. Thackeray's friend of twenty-five years' standing has drawn the long bow, for the edification of the general public, with a vigour and success altogether unapproached by the reporter for the special organ of the Spiritualists themselves.

Taking all the circumstances into account, we do not hesitate to express a very decided opinion that nine-tenths, or a larger proportion, of the physical manifestations of so-called spiritualism are neither more nor less than impudent frauds upon the credulity of the public; and we trust that some competent observer will undertake, with regard to them, researches analogous to those by which Sir John Forbes demolished the pretensions of the mesmerisers. We must fully admit the propriety of Professor Faraday's refusal to investigate such matters, based, as it was, upon the better occupation of his time; but there are many who might accomplish the task, as an amusement during leisure that would not, perhaps, be more usefully employed elsewhere. The mechanical and other contrivances in use are probably various, but there is little doubt that any one, possessing a moderate practical acquaintance with the applications of physical and mechanical science in the production of ordinary conjuring tricks, would very soon lay bare the more common methods of procedure; some of which, indeed, since this paper was written, have been graphically described and illustrated in the pages of *Once a Week*.

In the next place, we may remark that many of the statements made by the Spiritualists cannot possibly be true. We will not discuss the physical impossibility of table-lifting without the employment of adequate force, so long since pointed out by Professor Faraday, but will select, by way of illustration, such a sentence as the following:—

"We have heard the rappings upon the floor, as if produced by a crutch: in this case, a lady present informed the circle that that was the mode in which the spirit of her grandfather signalled his presence to her. . . . *All present saw exactly the spot whence the noise came, though no crutch or other means of making the sound was visible.*" —*Sp. Mag.*, No. 2.

Now it is very well known that the human senses are not so organized as to afford means of "seeing exactly the spot" whence a sound comes; nothing being more easy than to be deceived with regard to this very point. An exhibition of ventriloquism is a sufficient proof of this position; and a reference to the physiology of hearing will of course place the proof upon a scientific basis. Mixed up with a description of the alleged *facts* of spiritual intercourse, we have, therefore, a statement that is necessarily untrue, because it involves an impossibility; and it is difficult to avoid the supposition that a similar recklessness of assertion may characterize other parts of the same narrative.

Upon many points that would allow the application of tests, the language of spiritualists is too vague for refutation. For instance, they claim for trancee (*Sp. Mag.* No. 2.) a power of speaking about things, and in languages, lying beyond the sphere of their natural knowledge; and this claim is not sufficiently definite to be scrutinized. It is the most familiar of physiological facts that, in certain conditions of the nervous system, past sensory impressions, that need not have been understood, may be recalled; and in this way sentences of unknown or of forgotten languages, or scraps from scientific lectures or treatises may be brought back to the memory, and uttered, under the influence of suggestions or associations that would be inoperative in the normal state of the system.* So far as this goes, we may admit the

* The writer once attended a lady who, for two or three days was delirious after childbirth. She had been born in France, had resided there during infancy and facts alleged by the spiritualists, and deny their conclusions; somnambulism, either natural, hysterical, or artificial, being a sufficient explanation of such occurrences; and the only condition necessary to the so-called miracle being that what is uttered should have been *heard* by the speaker at some former period. If the mediums claim more than this, they must assume one of two positions: either that the spirit communicating speaks with the precise amount of knowledge that it possessed whilst inhabiting an earthly body; or else that the spiritual state involves an increase of knowledge with regard to physical science, and with regard to the deeds done, and the languages spoken, upon the world. Each of these hypotheses would admit of speedy and practical demonstration; and either of them, if found correct, would lead to results eminently advantageous to mankind. If the former alone were true, we should be able to obtain from mediums a vast mass of information concerning past occurrences that are imperfectly recorded or understood. Scholars would rejoice in the restoration of works now known to us only by precious fragments, Historians would terminate for ever their disputes about bygone facts. The departed miser would reveal his hoarded store; and the spirit of the victim would denounce the secret murderer, and point out the collateral evidences of his guilt. If the latter were true, the world would have realized, long before this, consequences which, but to think of, bewilders the imagination. Philosophers, painfully and laboriously seeking after truth, are surrounded, in every department of inquiry, by a dim circle of hypotheses, standing between positive knowledge and the unknown. If, by communication with higher intelligences, they could be freed from the doubts that these hypotheses imply, the progress of the last fifty years, vast as it has been, would speedily sink into comparative insignificance. Bishop South, in his noble sermon upon the character of Adam, uses the known capacities of manhood to illustrate the unknown. "All those arts," he writes, "varieties, and inventions, which vulgar minds gaze at, the ingenious pursue, and all admire, are but the reliques of an intellect defaced with sin and time. We admire it now, only as antiquaries do a piece of old coin, for the stamp it once bore, and not for those vanishing lineaments and disappearing draughts that remain upon it at present. And certainly that must needs have been very glorious, the decays of

---

early childhood, and spoke the language fluently; but was very slightly acquainted with French literature, and for several years had been away from French people, and from opportunities of French conversation. During her delirium, she sang French cradle songs continually, songs that she could not recal after her recovery. It is obvious that her memory was taken back to the sounds that she had heard in her nursery, and that she had long forgotten.

which are so admirable. He that is comely when old and decrepit, surely was very beautiful when he was young. An Aristotle was but the rubbish of an Adam, and Athens but the rudiments of Paradise." Surely we may pursue this magnificent parallel, and may learn from the vintage yielded by science in our time something of the possible scope of an intercourse that should realize what charlatans profess. We turn from such contemplation to the facts; and we find that modern spiritualism, as it is distinctively called, after six years' existence, has culminated in that brief elevation of Mr. Home, the very reality of which we have seen reason to call in question. Like its prototype of the time of Apollonius and of Simon, like mesmerism, divining rods, and table-turning, spiritualism has been absolutely barren of results. It has not added an iota to the sum of human knowledge, it has not settled a single doubt upon any subject. We judge it by its works—that great test applicable alike to doctrines and to men—and we find it entitled to a place with fifth-rate jugglery or gipsy fortune-telling.

The special forms of "physical manifestations" need not occupy much of our remaining space. Table movements, it is evident, may be produced either by unconscious muscular action during expectant attention, by the knee or foot of the medium or of an accomplice, or by machinery. The sources of the raps and other noises may be infinitely varied at the will of the performer. The appearance of spirit-hands has been greatly elucidated by the cartoons of *Punch;* and their feel by the following curious sentence—taken from the August number of the *Spiritual Magazine*—"the darker part of the room, and here arose a scene of indescribable confusion, but still producing feelings in no way unpleasant, though we knew not, *when we touched each other, who were spirits and who were fleshy human beings.*" ! ! It is, of course, possible that certain individuals may have spectral illusions—or subjective sensations as of hands; and here again the influence of expectant attention affords a sufficient clue to the phenomena, if such occur. On the same principle, luminous appearances may be explained; but these may be readily produced by most persons, in any dark room, without the intervention of a medium at all. They were made, by Baron Reichenbach, the basis of a perfect avalanche of rubbish about a so called od-force; a very complete examination of which may be found in the *Brit. and Foreign Med.-Chir. Review*, vol. viii. p. 378.

The terms od-force, odylic sphere, magnetism, magnetic fluid, electric fluid, &c., &c., form part of the machinery by which the spiritualists impose upon the credulity of the public. These terms are used as if they had definite and exact meanings, like water or milk; or as if the magnetic fluid or the electric fluid could be bought by bottlefuls of a druggist. The frequenters of the *séance* are probably not aware that the term "*fluid*," as applied by philosophers to electricity and magnetism, is nothing but a provisional name, and perhaps an unfortunate one, for agencies, the precise nature of which has not been discovered. There is no certain evidence of the existence of electricity as a distinct entity—as a fluid, and the word is only used as a convenient designation for the unknown cause of certain molecular changes in material bodies. Unfortunately, the public are not aware of this. The existence of the electric and magnetic telegraphs leads many an honest man to believe that, if he knows nothing about their respective "fluids" himself, others understand them thoroughly; and he accepts the pseudo-scientific jargon of the day as containing the complete theory of spiritual existence. It cannot be declared too loudly that these words, impudently put forward as the representatives of knowledge, are in reality nothing but the scanty coverings of the most utter ignorance; and that as employed in the *Spiritual Magazine*, and similar publications, they are absolutely without any intelligible meaning whatever.

It is impossible to conclude this part of the subject without some reference to the men by whom the so-called spiritualism is upheld. Leaving hired mediums out of the question, the most prominent names in the *Spiritual Magazine* are those of Dr. Ashburner and William Howitt. Of these gentlemen, the former was unpleasantly conspicuous in connexion with the practice of mesmerism in a metropolitan hospital. Since then, mesmerism has fallen into desuetude by force of utter worthlessness, and its some time champion appears as the apostle of a new delusion. Mr. Howitt is known to us only as having worked industriously for booksellers—a vocation more favourable to the memory and the invention than to the judgment. In their proper spheres, or even in any decorous and modest statement of their opinions, these persons are doubtless worthy of respect. But when Mr. Howitt epitomizes nearly the whole of the human race—namely, all who do not accept his hypotheses concerning spiritualism, under the fanciful appellations of Homo-Sus-Eruditus and Homo-Talpæus, and when both he and Dr. Ashburner raise their pigmy voices in railing against the gigantic intellect of Faraday, they put themselves beyond the courtesies of ordinary criticism. Our indignation at their failure in the respect due to the great philosopher of whom our age and country are so justly proud, must of course be largely tempered by a sense of the overpowering absurdity of the contrast that their assault suggests; but upon such criminals, however contemptible, justice must be done. "What," says Pope, "must be the priest, where the monkey is a god?" What shall we think of a trickery that has Home and Harris for its oracles—Howitt and Ashburner for the guardians of its shrine?

From the physical phenomena of spiritualism, we may now pass on to an examination of its literature, both descriptive and (professedly) dictated. The *Spiritual Magazine* is the most prominent example of the former, as the writings of Mr. Harris are of the latter.

The *Spiritual Magazine* need not detain us long, and only requires notice in order that we may point out a curious family likeness between the compositions of its various contributors. Through every diversity of style, through various degrees of knowledge, the imbecility of mind necessary to a belief in "spiritualism" makes itself apparent. From the fourth number we quote a portion of an article—italicising certain passages that are especially worthy of remark.

"The Rev. T. L. Harris, in his sermon of the morning of the 19th of February, 1860, said, as far as my memory serves me: 'Every flower, fruit, and tree emits into nature the best portion of its being—its essence. But who has seen the aromal essence of a flower? Who has beheld the essential form thus given off into the universe?'

"This question caused me to remember a curious circumstance which occurred some months ago at the residence of two relatives, neither of them sharing those spiritual beliefs which I hold dearer than my life. I will briefly relate the facts, for there are two. The first is as follows:—

"Another near relative and myself had visited my two lady relatives; and after tea, in the evening, a beautiful night-stock was placed on the table underneath a gas lamp with two burners, one of which only was lighted, with a green shade to throw the light down. As the fragrance of the flower diffused itself through the room, it was remarked by all of us, and I, not being familiar with the plant, was led to examine it more closely. And as I looked there seemed to be a floating mist rising from the flowers of the plant, which I immediately mentioned to my relatives; one of them, the one who accompanied me, and whose hand is used for spiritual communication, looked intently, and after a long time saw the 'smoke,' as we termed it, and then another of the party saw it—one of those who are incredulous on the subjects discussed in this magazine. But the fourth person did not see it.

"I have long noticed, it is here necessary to remark, that when I put my two forefingers nearly together, a spark invariably passes from the extremity of the right forefinger to the corresponding extremity of the left. Nor have my own eyes alone seen this; it has been seen by others, *and I have no doubt that under conditions, and if experiments be instituted on the point, this will be found common to all persons who, like myself, possess sanguine-nervous temperament.*"

The writer proceeds to relate that he approached his left forefinger to the stock, and that he immediately "perceived and felt" a flash (electric or odylic) pass to him from the flower. He appears to mean, moreover, that this power of "flashing" was continuous, and not exhausted by exercise; for he says, "The right forefinger produced *similar flashes*, but of less intensity," and he then breaks out into the following rhapsody—

"I regard this as a matter of science, *although* I do not for one moment doubt that spirit pervades all matter. The question for consideration is. What caused the flash *from* the flowers and leaves? It could not be with force of my own, as I was unprepared for the result; more probably—I throw it out only as an opinion—I had broken in upon the odylic sphere of the flower, which thus reacted upon the electro-odylic battery of my nervo-sanguine system. Cornelius Agrippa (whose three books on Occult Philosophy contain a mass of wonderful speculations upon nature, man, spirit, and God,) suggests the existence, *throughout his work*, of a subtile essence, sympathetic and antipathetic, between all things. *It* is a matter for investigation; and until a series of facts are eliminated by independent observers must remain uncertain."

It is possible that these pages may fall into the hands of some readers who are unaccustomed to scientific phraseology, and unacquainted with scientific facts. Such a possibility, and such only, will justify a brief commentary upon what is intended to be conveyed by this twaddle about tea and relatives.

In the first place, the sentence from Mr. Harris, the text of the whole affair, is absolute and unmitigated nonsense. If by *essence* be meant *perfume*—it is of course not true that every flower, fruit and tree emits it—because the vast majority are scentless. Neither is it true that this essence is anything invisible or recondite—as implied in the "Who has beheld?" &c.; for it consists—where it exists at all—of an essential oil, well known to chemists, and easily procurable in a separate form. If perfume be not intended, but something else, then that something must be either a definite chemical existence, such as oxygen—about which there is no mystery—or else a mere figment of Mr. Harris's diseased imagination. Then what is the meaning of "emits into nature"? What is "nature"? Plants emit their volatile essences into the atmosphere, which is part of "nature" in one sense of the word, just as the plants themselves are parts of it; but Mr. Harris seems to imply that the plant stands outside "nature" and throws something into it. Again, "the best part of a plant." What is "the best part" of a plant? and how does any one know that the part specified, whatever it may be, is, in any real sense, better than the rest? We apprehend that the only rational application of "best" to part of a plant, would be to the part most useful to man; and that this would be rational only in a very restricted sense—because the various parts are interdependent, and more or less necessary to each other. But such as our quotation are all the compositions of these would-be mystics, whether they call themselves Spiritualists or .nto Words capable of being used in a dozen different senses, and sentences which, when analysed, are seen to be without any particular meaning, make up the sum of the literature of the marvellous —as created by believers.

It is mentioned in all elementary treatises on botany, that the anthers of certain plants are elastic; and in bursting, cast out the pollen, or fructifying dust, in little clouds or puffs. Such an arrangement is chiefly found in erect flowers, having a style higher than their stamens; and it provides for the conveyance of the pollen to the stigma. We have here a sufficient explanation of the "smoke" from the plant, if, indeed, this were anything more than a subjective sensation; and the dry atmosphere of a gas-lighted chamber would be very favourable to the occurrence of the phenomenon in question.

It is obvious that the flash said to have passed between the fingers of the observer, could not have been electric. An electric spark would not be visible against gas light; and no such spark could be obtained in the manner described. An electric spark passes, whenever two bodies, differently charged with electricity, approach each other under favourable conditions. But, if it were possible for the right and left forefingers of an individual to be differently charged, the equilibrium would be restored through the unbroken continuity of the bodily tissues, and not by a spark passing through the atmosphere.

The other hypothesis suggested is, that the flash was "odylic." Now "odylic light" is a new name for a very old thing; that is to say for sensations, like those produced by light, but dependent upon changes in the sensorium itself, and not upon impressions from actual light falling on the retina. The optic ganglia of the sensorium are so organized as to convey, in their normal state, no impressions but those of light. It follows that almost any change wrought in them, and there may be many such, conveys the idea of light to the mind. Pressure on the eye produces a luminous spectrum. Blows on the head are well known to have the same effect. Expectant attention—*i.e.*, partial hypnotism, produces all sorts of luminous appearances, which, like those from blows or pressure, have no reality outside of the spectator. It is very likely, therefore, that the writer whom we quote may see "flashes" under a great many circumstances, all of them depending upon the state of his own nervous system: but, when he talks of other people seeing the same "flashes," he falls into the droll mistake of the Irishman, who, having had his head broken on a dark night, swore that he had recognised his assailant by the light that gleamed from his own eyes on receipt of the blow. It is of course possible that two independent flash seers may meet over the same pair of fingers, or the same flower-pot; but the identity of their flashes, unless pre-arranged, could be disproved by the simple device by which Daniel overthrew the testimony of the elders.

The remaining paragraph that we have transcribed is so utterly without meaning, that it may be useful, perhaps, as a mnemonic exercise. It ought to have been presented to the public for this purpose; as a dictation by the spirit of Foote, and as a continuation of the following:—

"So she went into the garden to cut a cabbage-leaf to make an apple-pie, and while she was there, a great she-bear popped its head into the shop. What! no soap—so he died; and she very imprudently married the barber, and there were present the Joblilies, and the Garuylies, and the Piccalillies, and the great Panjandrum himself, with the little round button at top, and they all fell to playing the game of catch as catch can, till the gunpowder ran out at the heels of their boots."

In papers of more pretension, in the *Spiritual Magazine,* we observe always the same confusion of thought, and the same obscurity of language. There is a Dr. Blank—professedly, and very possibly, M.D. Cantab.—who undertakes to record FACTS. He says, "I boldly claim for the facts I have here recorded that their evidence has been carefully tested by me and my friend X. Y." Now there is not the remotest indication of this testing in his descriptions; and we infer that Dr. Blank, like the author of *Stranger than Fiction,* has still to learn that very rudimentary piece of knowledge, the meaning of the word "fact."

We turn next to Mr. Harris, regretting that the space at our disposal compels us to treat him with a brevity that will accord with his merits, rather than with his pretensions.

The latter are nothing less than Apostolic. Himself a progressed "medium," and having passed through the physical and intellectual, to the *spiritual plane* of mediumship, he now says that he possesses—and he only—the power of discriminating between good and evil spirits—*e.g.*, between the spirit of Paul the Apostle, and an evil spirit personating that of Paul. With the delicious vagueness of his kind, he thus describes the powers with which he has been endowed:—

"Differenced, as to states, from the men of the present age, by means of an opening of the internal organs of respiration, which is continued into the external form, I inhale, with equal ease and freedom, the atmospheres of either of the three Heavens, and am enabled to be present, without the suspension of the natural degree of consciousness, with the Angelic Societies, whether of the ultimate, the spiritual or the celestial degree. It is impossible to inhale in this continued manner, from the celestial into the corporeal, without living among the angels. Inhaling the divine aura, by means of which respiration is continued, they exist in a waking reality of Divine Wonders. They enjoy, objectively, the vision of the Lord as a sun, illuminating, with the light of infinite truth, the expanses of the firmament. He manifests Himself in a verbal revelation through the word, which exists in every Heavenly Society. He is also made known to them in a direct appearance, and is transfigured before them in His Divine Human Form. Besides this, He speaks to them by an inmost voice which is audible in the sanctuaries of the breast. All of that tender intimacy which existed, in natural representatives, between our Lord and His disciples, during the period of His incarnation, is realized in His presence with the Angels. Having been finally intromitted into these three degrees of interior respiration, I was led upward, through the series of experiences of which the narration now ensues, that, by a pathway of easy and instructive transitions, I might approach the state of qualification to understand the arcana contained within the Celestial Sense of the Divine Word. At the close of these initiaments, as will be found in the context, it was my privilege to behold the Lord, whom I saw in His Divine Appearing, and who laid upon me the charge of receiving and unfolding such of those arcana of the celestial sense as are contained within this volume, and as will in due time be given to mankind in continuance of the labor which is here begun."*

Into the discussion of such claims as these, we cannot, of course, enter, but must content ourselves with looking at Mr. Harris's writings by the light of internal evidence alone. We are told by Mr. Howitt that the progressive nature of Mr. Harris's inspiration, and his constantly increasing enlightenment, produce changes in his views from time to time; and hence that we must not, in judging of his works, take occasional contradictions into account. Thus he has "*of late* broached the old doctrine of the perdition of certain souls; whereas, in 1855, he was a staunch universalist." We have more to say about Mr. Harris than the pages at our disposal can receive; and therefore we will leave the question of contradiction as we find it.

The "gifts to mankind" consist, at present, as far as we know, of poems, sermons, and the "Arcana of Christianity." These must be briefly noticed in their order.

The poems are of various kinds—sacred and profane. The following is the first verse of a hymn "given" by a spirit; and it is followed by three or four more of the same quality. The leading idea is one that no ballad-monger can degrade; but it was revealed to mankind before Mr. Harris's time, and we have only to do with Mr. Harris's treatment of it:—

* *Arcana of Christianity,* Part I. vol. i. p. 7.

"Oft when *storms of pain* are *rolling*,
And I cross *the fiery sea*,
Comes a voice, my heart consoling,
Jesus loves me,—even me."

With great submission to better judges, we think that the words italicised must have been a "gift" from the spirit of Mr. Robert Montgomery.

The next is a parody on "I dreamt that I dwelt in marble halls." Like the former, it is not original in conception, although strikingly so in treatment:—

"I dreamt that I dwelt in fiery halls,
With a serpent by my side;
She wound me in her venomed coils,
I had a demon bride.
She spoke with lips like snakes that stung,
And breath of *poisoned* flame,
You ruined me when my heart was young,
But I love you all the same.

My *heart* is now an *adder's lair*,
My *body* turned to *mould*,
I sit alone in dark despair,
Within the devil's fold.
For you I drank the cup of doom,
The harlot's sinful shame.
Come, clasp me in our fiery tomb,
For I love you all the same.

We drank of passion's cup, alas!
And reap what we have sown;
And see in hell's huge looking-glass,
*What beauties we have grown.*
You are the corpse of manhood now,
In spite of all your fame;
But, foul deceiver, hear my vow,
I love you all the same.

I'll make your heart my *dressing gown*,
And on it I will sit;
And, *like a rat*, your soul I'll drown
In hell's unfathomed pit.
Take back, take back, those fires of lust,
In wreaths of snaky flame,
I spit on thee, thou devil's dust,
But I love you all the same."

We offer these stanzas as a sufficient illustration of the surpassing beauty of "spirit-poetry." They are indebted to the "giver" for nothing but their precise verbiage—the rhythm being a parody, the idea that man's sin shall find him out, not being new, and the particular application of this idea being expressed, in every detail, by Swedenborg. If any reader wishes to institute a comparison between a "spirit" poem and a "fleshy" one, on the same subject, we recommend Campbell's ballad of the "Spectre Boat," as an illustration of the manner in which similar materials have been handled by an author of taste and genius.

The sermons are somewhat out of our direct line of criticism. They are what many people would call "fine compositions;" and are overloaded with tumid verbosity, and disfigured by such coinages as "*Familism,*" and "*Outmostly,*" until the actual meaning of the sentences can scarcely be discovered. When found, it is often like the "Emission of Essences" notion that we have already quoted; but, in general, the leading idea seems to be an approaching union of Christians of all denominations on a higher level of faith and duty than that of any single sect; and the means thereto, a general disregard of doctrinal distinctions. We do not ourselves feel that sound doctrine is a thing to be laid aside like an old garment; and we fail to see any evidence that these sermons are not the result of preparation and thought like the sermons of any other man. They are professedly "mediatorial," which means, in the slang of the spiritualists, that Mr. Harris is but the instrument of their delivery, and that he has no share in their production.

The "Arcana of Christianity" might have, as its second title, "The Spiritual Gulliver." Purporting to be a commentary on the first chapter of Genesis, it contains an account of Mr. Harris's visits to the Heavens, the Hells, and the planetary bodies—including some of the latter not known to astronomers. It contains, moreover, a particular history of the youth, education, decline, and fall, of the great enemy of souls—upon a planet now destroyed by reason of his crimes, and those of his followers. We cannot enter into any detailed examination of the volume, but must be content with stating general conclusions. Apart from any question of probability, we think the book is not to be received as truth, and for the following reasons:—

In the first place, there is an absence of that verisimilitude, arising from minute touches of description, by means of which the grace of fiction—

"Has power
To render things impossible believed;
And win them, with the credence of an hour,
To be for truths received."

We are told that the inhabitants of the moon are little people, like children of twelve years old, and "breathing from the abdomen;" that the men of Jupiter are of a sky-blue colour, dotted over with gold spangles, and so on; but we never stumble upon a sentence that conveys, by some little word, or turn of thought, the idea of a recital of actual experience. Safe generalities, sufficiently like the generalities of this world to remind us that imagination may alter, but cannot create, make up the bulk of the narrative. We find nothing quite unlike our present surroundings. We do not find any clue to the manner in which life is maintained upon those planets that are placed under physical conditions widely different, probably, from those of the earth; and we suspect that Mr. Harris, on his "natural plane," is not aware of the difficulties of this kind that are perceived by philosophers. However this may be, he contributes nothing to their removal; and the want of the details which such a spiritual traveller ought to relate, leads us to conceive that his book, as far as there can be a more or less in the matter, is less true than Gulliver's travels.

In support of this opinion, there is, moreover, something like positive evidence, if it be searched for. We must be content with a single illustration:

Swedenborg, whom, in all essential matters, Harris professes to confirm, describes the "angelic language" as surpassing the power of man to conceive, and as resulting from a direct relationship between sounds and ideas. Harris, describing an angel originally from the planet Saturn, writes, "He spoke in a language which seemed composed of liquid sounds, divested of all harshness." And yet we read, in the same volume: "There is a planet beyond the orbit of Neptunus, as it is externally styled, *which is called Polyhymnia* BY THE ANGELS. If it were Πολύμνια, or even Polymnia; but Polyhymnia! and by the angels! It would not perhaps be inconceivable that an angel should speak Greek to Mr. Harris; but anglicized Greek is too much of a good thing; and the words quoted certainly imply that the planet is called Polyhymnia among themselves. We read also of another planet, "between the sun and Mercury, *called Corona*;" and why one planet should have a name that is half Greek, and another, one that is entirely Latin, we must confess ourselves wholly at a loss to understand.

There is another objection to Mr. Harris's writings, one that it is painful to urge, and yet not possible to ignore. The *Arcana of Christianity* abounds with passages concerning sexual love, earthly, planetary, angelic, and demoniac, until, indeed, directly and indirectly, the relations between the sexes furnish the most prominent subjects in the volume. Some of the passages are only voluptuous; others absolutely filthy. In Polyhymnia "the yieldings of the bride are in obedience to the descent of a direct influx from the Lord Himself; but more than this I am not permitted to narrate. The bliss of nuptials is prolonged to the close of life, when it gently merges into immortality. The wife is as fond at ninety, about which period translation generally takes place, as in the sabbatic rapture of the bridal dawn. They have no name for coldness, because it is never experienced, nor are the wives ever satisfied but to rest in their husband's embraces, &c., &c." In this world "those unfortunate creatures who prostitute their bodies for gain, are attended by demons, who live, *as to their subtle organic parts, so for as they are magnetically extended into the subtle realms of nature, by absorbing into their bodies the degraded substance which otherwise might become inwrought into the corporeal tenements of little children.* Hence it is that after a period they (qy. the demons) are able to become mothers no more." We cannot give any other examples of this balderdash; but all practical physicians will agree that such an excessive prominence of erotic ideas throws great light upon the condition of the author of the work; and forcibly suggests the means by which that condition has been produced.

It is curious, moreover, that similar thoughts and feelings were commonly manifested by those persons who, in the sixteenth and seventeenth centuries, abandoned themselves to the delusions of pretended witchcraft and sorcery. The explanation may be, however, that the great majority of these unfortunates were crazy; and therefore, as sexually excited lunatics are always a numerous class, and as demonomania was, at that time, the prevailing type of insanity,—so the frequent combination of the particular depravity with the particular hallucination may be ascribed, perhaps, to necessary coincidence alone. Whatever the explanation, there can be no doubt about the facts; for the accusations against the supposed witches and sorcerers always imputed, and their confessions under torture always admitted, actions prompted by morbid sexual desire. For instance, the following passage is cited by M. Figuier from the official abstract of the confessions of persons condemned for sorcery and witchcraft, in 1609, by MM. Espagnet and Delancre, commissioners appointed by Henri IV. to investigate and punish an outbreak of demonomania in the neighbourhood of Bayonne. The original is given in the French language; but, from considerations which Mr. Harris ignores, we have thought it best to present it to our readers in Latin.

"Modum reperierunt quo uxores amplexibus maritorum abripiant; et, sancto sacroque vinculo conjugii violato, mœchantur et fruuntur coram ipsis maritis, qui, velut statuæ immobiles, sui honoris damnum conspexerunt sine potestate ut tanto sceleri obstent. Ipsa conjux, muta, coactâ silentiâ superata, frustra auxilium mariti implorat;—maritus, fascinatus, impotensque, oculis apertis, decussatisque brachiis, suum dedecus aspicere compellitur.

"Saltare inverecundissime, epulari fœdissime, cum demoniis copulare, blasphemare turpissime, ulcisci insidiose, omnibus libidinibus horrificis subsequi, pædicare flagitiosissime, bufones et viperas de voluptate animi alere, omnigena rara aconita habere, caprumque graveolentem amare et amplecti, feruntur."

The conclusions that force themselves upon us, after this hasty review of the physical and literary phenomena of spiritualism—may be summed up very briefly. We think that these phenomena are the results of three elements, hypnotism, fraud, and delusion; and that these have all been present in almost every *séance* of which we have seen any record. It is clear from internal evidence that the works professedly dictated by spirits contain nothing that might not emanate from the brain of the medium; and that their lofty pretensions cannot for a moment be sustained in the face of criticism. We do not feel called upon to attempt to separate mere folly from actual fraud, or to indicate on which side of the boundary line our personal convictions would induce us to place this or that prominent believer. We say farewell to the so-called Spiritualists as a whole; wishing the rogues more honesty, and the dupes more sense.

---

